# Blood Group Typing: From Classical Strategies to the Application of Synthetic Antibodies Generated by Molecular Imprinting †

**DOI:** 10.3390/s16010051

**Published:** 2015-12-31

**Authors:** Adnan Mujahid, Franz L. Dickert

**Affiliations:** 1 Department of Analytical Chemistry, University of Vienna, Währinger Straße 38, A-1090 Vienna, Austria; adnanmujahid.chem@pu.edu.pk; 2 Institute of Chemistry, University of the Punjab, Quaid-i-Azam Campus Lahore 54590, Pakistan

**Keywords:** ABO-blood group typing, agglutination, synthetic receptors, molecular imprinting

## Abstract

Blood transfusion requires a mandatory cross-match test to examine the compatibility between donor and recipient blood groups. Generally, in all cross-match tests, a specific chemical reaction of antibodies with erythrocyte antigens is carried out to monitor agglutination. Since the visual inspection is no longer useful for obtaining precise quantitative information, therefore there is a wide variety of different technologies reported in the literature to recognize the agglutination reactions. Despite the classical methods, modern biosensors and molecular blood typing strategies have also been considered for straightforward, accurate and precise analysis. The interfacial part of a typical sensor device could range from natural antibodies to synthetic receptor materials, as designed by molecular imprinting and which is suitably integrated with the transducer surface. Herein, we present a comprehensive overview of some selected strategies extending from traditional practices to modern procedures in blood group typing, thus to highlight the most promising approach among emerging technologies.

## 1. Introduction

Red blood cells (RBCs) or erythrocytes are differentiated from each other on the basis of their surface antigen structures. It was Karl Landsteiner who first discovered [1] the ABO blood group (BG) system in 1900 and rhesus (Rh) BG later [2]. Today, safe blood transfusion is greatly attributed to the pioneering efforts of Karl Landsteiner on human BGs. In general, more than three hundred genetically-different BGs [3] have been determined; however, the ABO and Rh BG system has fundamental importance in transfusions. In clinical laboratories, it is standard procedure to test for BGs A (containing only A antigens), B (containing only B antigens), AB (having both A- and B antigens), O (neither A nor B antigens) and Rh (giving information about the presence or absence of Rh antigens). However, unexpected antigens could be present in some individuals that may not have particular RBC antigens. Nonetheless, certain antibodies are expected to be present in the blood serum of these individuals. For successful and safe blood transfusion, it is important to have knowledge about the compatibility of donor and recipient BGs, *i.e.*, ABO and Rh. An incompatible or mismatched transfusion would make blood clump or agglutinate, which could lead to serious consequences and sudden death, as well. Therefore, a suitable cross-matching test between the intended donor and the patient is highly recommended and is a part of routine clinical analysis [4].

In general, the practice of analyzing RBCs to identify the nature of antigens [5] present in a blood sample is named BG typing. Principally, BG typing refers to a distinct chemical reaction between specific antibodies and BG antigens to monitor agglutination or blood clamping. In this way, the desired information about the nature of those particular antigens can be obtained. There is a wide range of various analytical tests and tools for BG typing [6], including some classical ones, such as tube or slide tests, whereas microplate and gel centrifugation are relatively modern-day methods [7] for blood typing. In addition, nucleic acid amplification techniques are feasible, especially in those cases where BGs are difficult to identify by serological methods. The ultrasound back scattering strategy [8] was also exploited for blood typing to monitor the agglutination reaction. This method offers suitable quantitative information about the agglutinated particles at an early stage and also explains the effect of shear stress on agglutinate equilibrium. Recently [9], a modern strategy was explored where elastic scattering of laser radiation is followed by digital imaging for determining human BGs. This hybrid acousto-optical approach has demonstrated high resolving power in monitoring sedimentation of RBCs and their agglutinates [10]. Polymerized chain reaction with sequence-specific priming (PCR-SSP) has also been put forward by researchers for molecular genotyping [11] of human BGs. Some cutting edge technologies aim at the determination of rare or weak alleles of BG; however, in both classical and advance techniques, there is a compromise between sensitivity, time of analysis and ultimate cost of that particular test. Furthermore, in some techniques, highly-trained personnel are required for interpreting blood typing analysis reports. Therefore, it is difficult to prefer a single testing method that offers sensitive and speedy results at a relatively low cost.

Miniaturized chemical sensors [12] are receiving increasing attention for their sensitive and selective response, rapid results and with the feasibility of in-field measurements [13]. These sensors can perform efficiently in complex mixtures [14] and, therefore, have found numerous applications in many fields, e.g., biotechnology [15], environmental monitoring [16] and clinical diagnosis [17]. In the design of biochemical sensors, the interfacial part could range from natural antibodies to synthetic receptor materials [18]. Although natural antibodies are greatly selective and specific in their binding, they suffer from regeneration problems, storage stability, are highly expensive and their derivation is not straightforward. In this scenario, synthetic antibodies crafted by molecular imprinting [19,20,21,22] offer comparable sensitivity and selectivity as that of natural competitors, are easy to synthesize and can be reused for several analyses with adequate stability. These features make synthetic antibodies exceedingly suitable candidates in modern biosensor [23] design and especially for blood typing.

In this article, we present a concise overview of some selected strategies in blood typing extending from classical methods to state-of-the-art modern biosensor devices equipped with synthetic antibodies as generated by molecular imprinting. Additionally, some latest developments in this area will also be highlighted briefly. The proposed methods will be discussed in view of their sensitivity, specificity, analysis time, cost and their applicability for in-field applications.

## 2. Classical Strategies in BG Typing

In routine clinical analysis, there is a wide range of established procedures and practices for blood typing, where nearly all of them deal with the formation of agglutinates. Even though some of these classical methods are not highly sensitive, nonetheless, they still hold importance in ABO grouping tests. There is a wide range of blood typing techniques [6], which differ from each other in terms of sensitivity, reagents and equipment required, the time of operation and throughput analysis. Herein, we describe some general approaches of blood grouping along with their inbuilt advantages and drawbacks.

### 2.1. Slide Method

The slide test is relatively the least sensitive method among others for BG determination, but due to its prompt results, it is very much valuable in emergency cases. In this method, a glass slide or white porcelain support is divided into three parts, as for each part, a drop of donor or recipient blood is mixed with anti-A, anti-B and anti-D separately. The agglutination or blood clumping pattern can be visually observed from which the ABO and rhesus D (RhD) type of blood can be determined. The test completes in 5–10 min and is inexpensive, which requires only a small volume of blood typing reagents. However, it is an insensitive method and only useful in preliminary BG matching for getting an early result. The test cannot be conducted for weakly or rarely reactive antigens from which the results are difficult to interpret, and additionally, a low titer of anti-A or anti-B could lead to false positive or false negative results. Although the slide test [6] is useful for outdoor blood typing, it is not reliable enough for completely safe transfusion.

### 2.2. Tube Test

In comparison to the slide test, the tube test is more sensitive and reliable; therefore, it can be used conveniently for blood transfusion. In this method, both forward (cell), as well as reverse (serum) grouping is carried out. The forward grouping suggests the presence or absence of A and B antigens in RBCs, whereas reverse grouping indicates the presence or absences of anti-A and anti-B in serum. In forward grouping, blood cells are placed in two test tubes along with saline as a diluent media, and then one drop of each anti-A and anti-B is added separately in these samples. These tubes are subjected to centrifugation for few minutes, and then, the resultant matrix is gently shaken for observing agglutination.

For precise blood grouping, the two tubes can be categorized according to the extent of blood clumping. The purpose of centrifugation is to ensure enhanced chemical interactions, particularly for weaker antibodies to react, thus leading to agglutination. Some potentiators could also be added to promote the agglutination; moreover, the long incubation of tubes also favors these reactions without drying of the test samples. In a similar fashion, reverse grouping can be performed, as here, the blood serum is treated against RBC reagent groups of A1 and B, and the subsequent agglutination pattern is monitored. The grading of agglutinates in both forward and reverse grouping is useful in comparing the difference in the strength of hemolysis reactions. In general, the tube method is much more sensitive than the slide test and requires a low volume of reagents, and some unexpected antigens can also be detected; therefore, it is a better option for safer transfusions. However, in infants, reverse grouping is somewhat difficult to perform, since they produce insufficient amounts of antibodies to be determined.

### 2.3. Microplate Technology

Among classical methods, microplate technology is a further step towards more sensitive and fast blood typing analysis with the feasibility of automation. In this technique, both antibodies in blood plasma and antigens on RBCs can be determined. Typical microplates consist of a large number of small tubes that contain a few µL of reagents, which are treated against the blood samples. Following centrifugation and incubation, the subsequent agglutination can be examined by an automatic read out device. The microplate technique was first introduced in early 1950s; however, since then, considerable developments have been made in the design to improve the performance. The foremost advantage of microplate technology is its fast response, low reagent volumes and high throughput analysis. Apart from microplates, gel cards or strips can also be used for blood grouping in modern immunoassay machines.

### 2.4. Column/Gel Centrifugation

Column agglutination technology or gel centrifugation is a relatively modern approach that has gained substantial interest in ABO blood grouping, as it intends to establish a standard procedure for quantifying cell agglutination. Here, the column is made of small microtubes that contains gel matrix to trap agglutinates. Blood serum or cells are mixed with anti-A, anti-B and anti-D reagents in microtubes under controlled incubation and centrifugation. The gel particles trap the agglutinates, whereas non-agglutinated blood cells are allowed to pass through the column. The analysis time can be reduced by using glass beads in place of gel material, since in this way, faster centrifugation speeds can be achieved, which leads to rapid results. This technology is sensitive, straightforward and relatively easy to operate for less trained personnel.

## 3. Synthetic Receptors Generated by Molecular Imprinting for ABO Blood Typing

Synthetic receptors [24,25] crafted via molecular imprinting have shown considerable impact in designing advance biosensors. The utmost importance of molecular imprinted polymers (MIPs) is due to their substantial bio-recognition competency and their high chemical stability. The synthesis of these biomimetic materials [26] is straightforward and relatively easy, which altogether results in its low cost. They can be stored at ambient conditions for several months, keeping the same recognition characteristics, whereas the lifetime of natural antibodies is limited, and they need to be stored under controlled environments. These features add value to molecular imprinted receptors and, therefore, are the main driving forces in switching from natural antibodies to synthetic polymers. For instance, the price of a typical natural antibody is in the range of $100–$1000 for 1 mg, whereas MIPs for a variety of different targets can be obtained in the range of $0.1–$0.5 [27]. This clearly indicates the cost benefit analysis in using MIPs in contrast to natural receptors. Realizing the potential advantages of MIPs, they have been utilized in a variety of fields, such as chiral separations [28], immunoassays [29], drug delivery systems [30], chemical biosensors [31] and many others. In the context of blood typing, MIPs can be designed according to the target shape, as the templating process [32] results in analogous cavities for analyte binding. Conventional bulk imprinting [33] deals with the smaller size targets, whereas for large bio-macromolecules [34], relatively modern techniques, like surface [35] or epitope imprinting [36], are preferred. Although in the bulk method, the number of interaction sites is larger, the diffusion pathways are long, which leads to prolonged response time; additionally, the complete release of target species is somewhat difficult, which ultimately reduces the regeneration of receptor sites. Contrarily, surface or epitope imprinting strategies offers shorter diffusion corridors for analytes with complete reversibility, which leads to prompt response and improved reusability.

**Figure 1:**
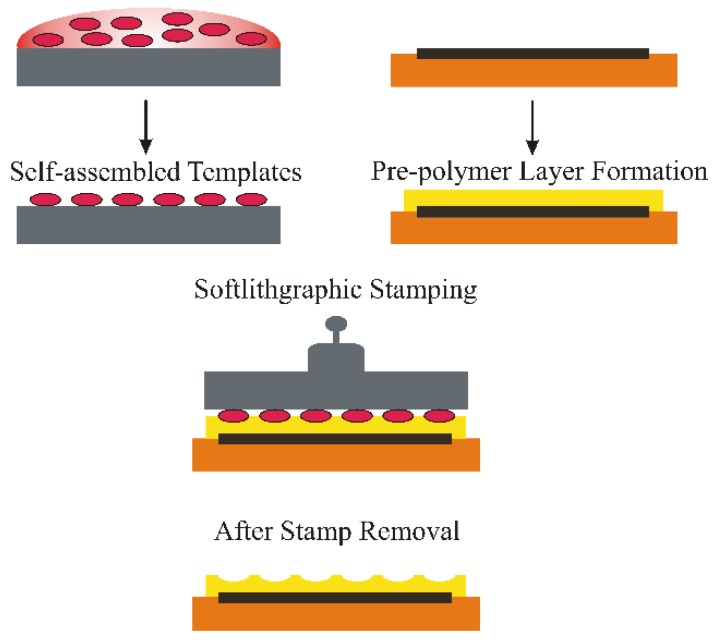
Schematic representation of the soft lithographic surface imprinting process.

In surface imprinting, the soft lithographic technique [37] can be applied to structure the polymeric layer material, especially for sensitive and fragile bio-species. Moreover, soft lithographic imprinting [38,39] of the target analyte can be done directly on the transducer device surface, which results in appropriate integration of the sensor layer with the device. A suitable densely-packed template stamp is pressed on an already spin-coated pre-polymer surface and allowed to cure under mild temperature and humidity for some time. This initiates the oligomer chains to self-organize [40] around the template and fixes their positions. After removing or washing the template cells, replicate cavities are formed in accordance to the shape of target species. The washing is done with mild solvents that eradicate only the template molecules and do not disturb the polymer structure. The imprinted structures are capable of recognizing a target form their shape, as well as chemical interactions. A schematic representation of the soft lithographic surface imprinting methodology is illustrated in Figure 1.

### Surface Imprinting of Erythrocytes

**Figure 2:**
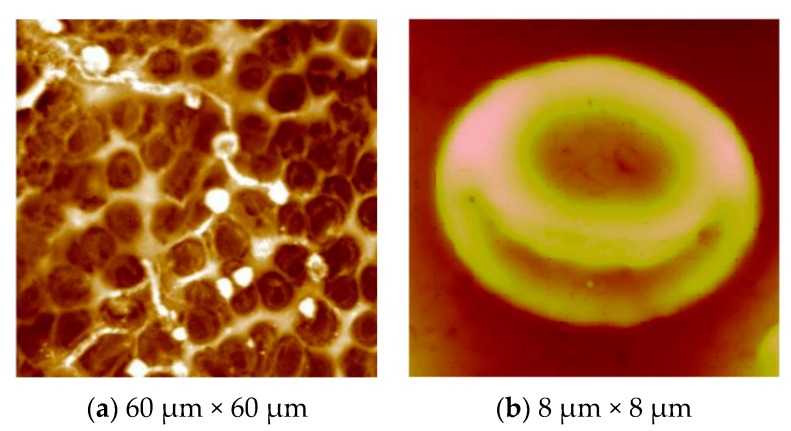
(**a**) AFM images of erythrocyte imprints on the polyurethane layer (white color, some cell fragments on the surface); (**b**) an image of a single erythrocyte imprinted on the polyurethane surface has been captured; adapted from [41,42] respectively.

This technique can be applied to imprint erythrocytes on a pre-polymer surface. The designing of stamp generation is relatively different, as the erythrocytes are a soft cell species and require special purification and pretreatment steps to discard added substances. The basic principle of the erythrocyte stamp was derived from the osmotic effect, *i.e.*, osmolysis, where RBCs are treated with isotonic NaCl solution. First of all, the erythrocyte concentrate is washed three to four times with isotonic NaCl, *i.e.*, 0.9% at a low temperature of 4 °C to avoid any degradation. This solution is centrifuged at 3000 rpm for 10 min, thus recollecting blood cells and removing the supernatant. After each washing step, the process is repeated in the same way to obtain the desired purity of RBCs. A suitable volume of resultant erythrocytes, *i.e.*, 3 µL of packed cells, can be deposited on a polydimethylsiloxane (PDMS) surface and spun off at 2000 rpm to develop a monolayer of RBCs. The use of glass instead of PDMS could lead to tight adhesion of blood cells, which makes the complete removal of the template difficult. The erythrocyte stamp is pressed over an already spin-coated pre-polymer surface and allowed to cure overnight at a suitable temperature. In the case of free radical polymerization, the erythrocytes’ molded pre-polymer matrix is placed under UV light at room temperature. After polymer curing, the templates can be removed by washing with hot water. Now, this surface contains the imprints of RBCs of exactly the same shape, and it can be viewed by atomic force microscopy (AFM). One such image is displayed in Figure 2, where the polyurethane surface has the impression of the RBCs. The soft lithographic imprinting [41,42] procedure is interesting and promising in order to transfer the structural details of bio-species onto a synthetic polymer interface. The imprinted polymer surface can reversibly reincorporate the template cells. It is interesting to note that the shape and size of all of the blood groups is the same; nonetheless, synthetic antibodies derived from MIPs can differentiate blood groups on the basis of the difference in their cell surface.

In view of sensor measurement for blood typing, the beneficial aspect is that imprinting can be made on a polymer surface that is already coated on the transducer device. Therefore, no problems exist concerning polymer transducer integration [43], and thus, sensor measurements can be made straightaway after template removal. The purpose of using silicone polymer is to avoid any damage to the shape of RBCs, as they are flexible in nature, and using a rigid substrate, like glass, as a support material is not useful. Furthermore, glass stamps could lead to tight adhesion of template cells on the polymer surface, which is sometimes difficult to wash out.

**Figure 3:**
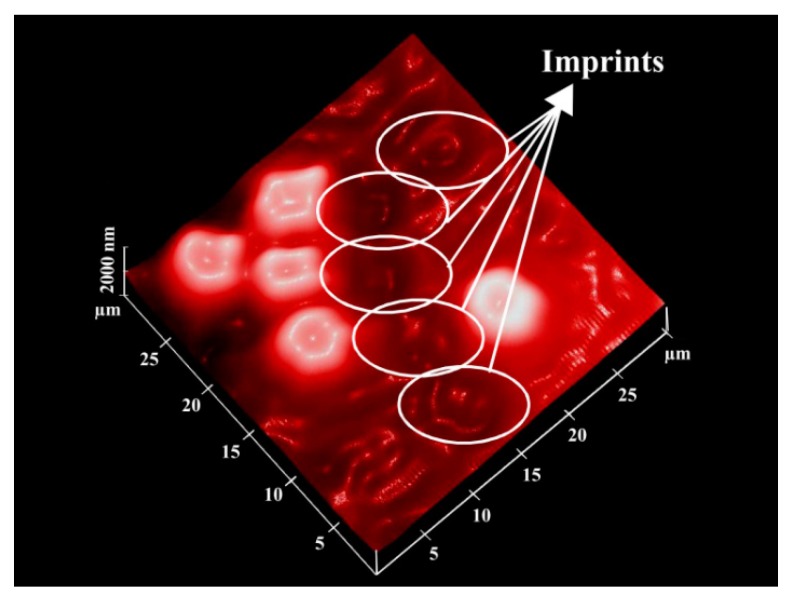
AFM image of the erythrocyte imprinted polyvinylpyrrolidone surface; adhered erythrocytes are shown, whereas white circles indicate the cavities formed after washing away erythrocytes; adapted from [44].

As the erythrocytes are a fragile species and need extra care during the stamping process, a modified version [44] of cell surface imprinting can be adopted. After thoroughly washing of erythrocytes as described above, blood cells are suspended in 1% (v/v) solution of 1,5-pentadial in isotonic PBS buffer of pH 7.2 for three min. The purpose of using 1,5-pentandial is to modify the erythrocytes’ cell surface, as this leads to crosslinking of RBCs. In this way, blood cells become more rigid and robust, which can then undertake the imprinting process, as shown in Figure 3. Furthermore, the imprinting density is also enhanced, since there are more interaction sites compared to the previous imprinting method. This is also beneficial in improving sensitivity. After modification, cells are washed and diluted accordingly to develop a monolayer on the support. This stamp is coated with PDMS and placed in a vacuum for one hour in order to remove air bubbles. Polymerization is completed at room temperature after a certain time, and by sonication, the stamp can be removed. Casting prepolymers on these patterned materials results in the generation of plastic or artificial cells, which are more robust than natural blood cells and, therefore, can be more useful for structuring polymer surfaces. The artificial cell stamp can be called the master imprinting stamp. This brings the possibility of crafting synthetic cells of the same shape as that of native cells.

The imprinting of blood cells can be directly made without using any stamping method. In such a practice, a pre-polymer mixture is first coated on the transducer surface, and after, packed cells are spread or deposited over this pre-polymer layer by spin coating. Now, the polymer can be hardened thermally or by a photochemical reaction under UV light, *i.e.*, using a free radical polymerization system. At the start, the polymer layer is relatively less viscous, and cells are easily deposited without using any support material, and during the course of the reaction, oligomer chains are self-organized around the cells. The important point in this methodology is that biological cells, *i.e.*, RBCs, are chemically sensitive, and before their degradation in matrix, the polymer should be cured. Therefore, considering this point, rapid polymerization is favored immediately after cell sedimentation. Imprinted cells can be washed from the polymer surface simply by rinsing with water.

## 4. ABO Blood Group Typing

### 4.1. Synthetic Receptors for ABO Blood Group Sensors

Biomimetic surfaces [45] as designed by soft lithographic imprinting are highly useful for selectively recognizing erythrocytes. For developing a typical sensor, surface imprinted layers are combined with a suitable transducer, such as acoustic or mass-sensitive devices. The foremost advantage of using acoustic devices [46,47] is that they are universal transducers, because mass is the fundamental property of any analyte that can be determined. Moreover, they are remarkably sensitive, which makes them highly valuable while detecting low analyte concentrations. Quartz crystal microbalance (QCM) and surface acoustic wave (SAW) resonators are typical examples of mass-sensitive devices and are widely used for developing chemical sensors.

**Figure 4:**
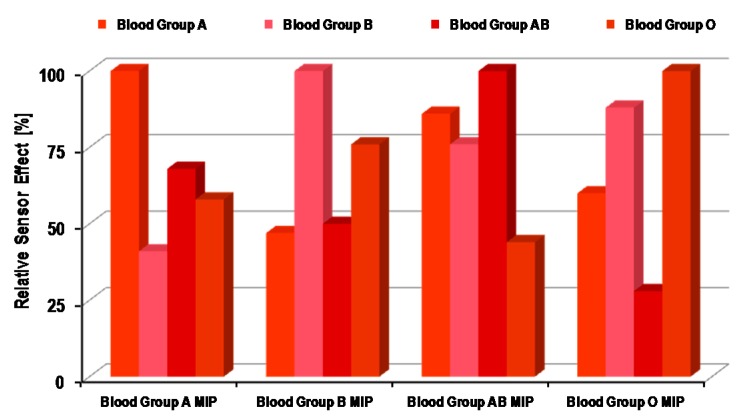
Relative sensor effects of surface imprinted polyurethane layers for ABO blood groups; in each case, molecular imprinted polymer (MIP) layers showed the highest response for those blood groups that were used for their surface imprinting; adapted from [48].

The integration of the layer material with the transducer is straightforward, as it was previously mentioned that the pre-polymer matrix is spin coated on QCM before erythrocyte imprinting. Figure 4 represents the relative sensor responses of four different polyurethane layers for the ABO BG system [48]. The surface of each polyurethane layer was imprinted with BGs A, B, AB and O separately. The sensor response of each layer for all four BGs is summarized in this graph. As one can see, each polyurethane layer exhibits the highest sensor signal for the BG that was used as the template during the surface imprinting of that polymer layer. For example, the polyurethane layer imprinted with BG A shows the maximum response for this BG during measurements, whereas the sensor response is relatively less for other BGs, *i.e.*, B, AB and O. For the other three polyurethane layers, a similar trend was observed. It is interesting to note that all of these BGs have nearly the same size, *i.e.*, in the range of 6–8 µm, but have different surface chemistry. Therefore, the imprinted polymer surface recognizes them by the difference in their surface groups, rather than by simple shape. The imprinted polyurethane films precisely incorporate target analyte cells by chemical interactions between erythrocyte surface antigens and polymer functional groups, thus providing chemical fitting. Concerning surface antigens of the ABO system, the most ample antigens are carbohydrates on the glycocalyx surface of erythrocytes. Terminal carbohydrate groups on BG O antigens, such as N-acetylgalactosamine and galactose, develop differentiation between BG A and BG B antigens, respectively.

**Figure 5:**
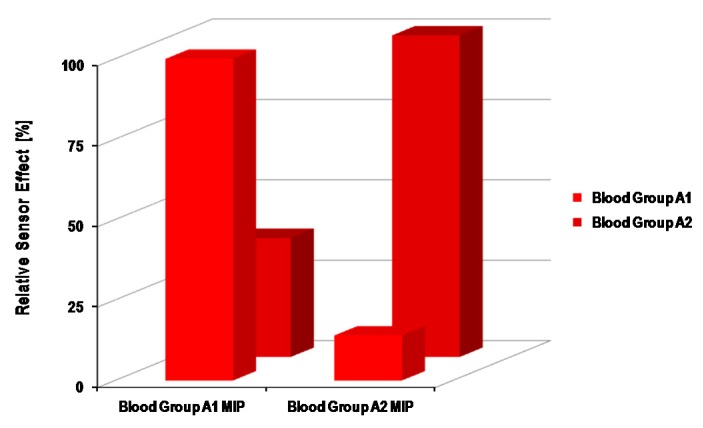
Relative sensor effects of the surface imprinted polyvinylpyrrolidone layer for sub-blood groups A1 and A2; as is obvious, the sensor response is highest when the analyte and template both belong to same type of erythrocyte; adapted from [49].

The extent of the difference in surface antigens is also evident from the sensor responses of imprinted polyurethanes films. For instance, the surface antigens of BG B are more similar to BG O, and this can be seen from their respective sensor effects. The polyurethane layer imprinted with BG O showed the second highest sensor response for BG B after the response of BG O. Additionally, the same trend can be observed while having a polyurethane layer imprinted with BG B. This indeed confirms that recognition is based on the matching of surface antigens with polymer functional groups. The driving forces between polymer and cells are mainly non-covalent interactions, such as hydrogen bonding. In order to make such associations more pronounced, excessive amount of crosslinkers are added, e.g., hydroxyl groups in the case of polyurethanes. As the increased proportion of such functional groups in the polymer matrix would develop suitable interactions with carbohydrates on the blood group cell membrane, which leads to improved recognition, therefore it can be concluded that the decisive part in getting high selectivity is the chemical functionalities between the analyte and receptor surface. Although imprinted surfaces generate cavities of complementary shape and dimensions, however they do not accomplish adequate selectivity when working with analytes of the same shape. MIP-based receptors can be tuned to differentiate between sub-blood groups, e.g., A1 and A2, which are predominant sub-blood groups in the ABO system. The basic difference between A1 and A2 is the antigen density on the erythrocyte cell surface. A1 has 0.81 × 10^6^–1.17 × 10^6^ A antigens per cell, whereas A2 has 2.4 × 10^5^–2.9 × 10^5^ A antigens, explaining the quantitative difference between them. It has been reported that A2 antigens possess distinct H-activity. Some natural antibodies can agglutinate only the A1 sub-blood group and not A2, which makes the foundation of the qualitative difference between these two sub-blood groups and also suggests a distinct antigen pattern on the sub-blood group cell surface. In general, the differentiation between A1 and A2 is very complex and challenging. Therefore, developing a blood group typing sensor [49] that can differentiate between A1 and A2 sub-blood groups would be of high value and importance. Such a sensor had been realized by using surface imprinted polyvinylpyrrolidone (PVP) as the receptor material coated on QCM, since PVP is more favorable than polyurethane layers in achieving high selectivity. The relative sensor effects of A1 and A2 imprinted PVP layers for sub-blood groups are shown in Figure 5.

From this result, it is evident that each PVP layer shows the highest sensor effect to that sub-blood group that was used as the template during imprinting. The relative response of each layer is at least three-times higher for the target sub-blood group as compared to the competing sub-blood group. As earlier, it was mentioned that all ABO BGs possess the same size, but have a different cell surface, here, in this case, sub-blood groups A1 and A2 share the same glycolipids cell interface, since they both represent the same blood group of the ABO system. The only difference is the amount of antigens on the respective cell surface. Therefore, such a high selectivity can be explained in view of the difference in cell surface modeling. The imprinted PVP layers recognize the A1 and A2 blood groups from their specific antigen pattern on their surface, since the imprinted cavities possess the desired configuration at binding sites, which accomplish such a selective response. There is a principle difference in the binding mechanism of natural antibodies and the synthetic receptor as prepared by molecular imprinting. The natural receptor recognizes targets by binding only at specific regions and, therefore, could lead to similar effects for two different sub-blood groups. Contrarily, the MIP-based receptor recognizes targets as whole cell species and, thus, can discriminate between sub-blood groups, as shown in Figure 5. As the structure of the erythrocyte cell surface is quite complex, *i.e.*, composed of diverse glycoproteins, glycolipids and a mixture of different antigens, therefore it is more favorable to distinguish RBCs as a whole unit, rather than identifying them from a specific position. Thus, MIP-based chemical sensors are exceedingly valuable, because they not only exhibit high selectivity, but also give complete information about cell binding.

Brouard and coworkers [50] developed an optical nanobiosensor based on metal silica fluorescent nanoparticles for BG genotyping. Silver nanoparticles were taken as the supporting core material, and on its surface, an eosin-functionalized silica shell was grafted. A polymer-based transducer was integrated to electrostatically bind with negatively-charged probes. The developed nanosensor can be used for correct identification of blood genotypes from non-amplified DNA samples, as this was compared to standard PCR assay results.

### 4.2. Natural Receptors for ABO Blood Group Sensors

Sensors containing natural antibodies as the receptor interface have also been developed for blood typing. A surface plasmon resonance (SPR) sensor was designed [51] for blood typing by immobilizing monoclonal antibodies, *i.e.*, anti-A or anti-B, on a thin layer of gold. These two antibodies respond to their corresponding antigens, which leads to blood typing. The detection principle is simple; for instance, if a sample containing BG A flows through the measured cell, the SPR sensor chip coated with anti-A will respond to it, whereas the chip immobilized with anti-B will not show any change. Additionally, in the same way for BG B, anti-B will respond to it, while anti-A will not exhibit any change. In the case of BG AB, both anti-A and anti-B chips will respond to it; however, for BG O, anti-A and anti-B would not demonstrate any response. The authors proposed this setup as a fast and sensitive method for examining ABO blood group typing, which can be applied to both saliva and blood. Another SPR sensor approach was also reported for the recognition of blood group-associated antigens on the erythrocyte surface. Blood group-specific antibodies, *i.e.*, IgM, were covalently fixed [52] to a dextran matrix and followed the consequent blood cell binding, which was quantified. Using blood group A-specific antibodies showed substantial binding for BG A only, whereas the non-specific binding of BG B and O was not observed. In a similar way, anti-B leads to specific binding of BG B simply and not for BG A or O. Using the SPR sensor, a detection limit of less than 0.33 × 10^9^ RBC/mL was reported, having linear standard curve in the concentration range of 0.33 × 10^9^–2.40 × 10^9^ RBC/mL. It was experienced that the sensor can be regenerated for repeated measurements by using 20 mM NaOH solution, as this leads to elute-bound erythrocytes. Despite such a restoration protocol, the activity of antibodies was not fully reestablished and was a major limitation for improving the binding cycles for blood group typing. In another report [53], again, an SPR setup was established for quantifying IgG anti-A and anti-B titers in about 15 min, which was a relatively shorter time than ELISA and flow cytometry [54]. However, in view of the number of samples measured at once, SPR is relatively less favorable than ELISA or flow cytometry.

Natural receptors, such as IgG, were combined with QCM for blood group typing [55]. The antibody-antigen reactions are carried out by exposing QCM to 20 µL of a 0.1% blood sample diluted with 10 mL phosphate buffer (10 mM) at pH 8. The quantification of the results can be made by monitoring the frequency shifts. It was also claimed that the QCM sensor can be recycled by treating with glycine buffer solution at pH 3. In another study [56], a quartz immunosensor was developed for ABO blood group typing. As the blood plasma is a highly viscous liquid, therefore the sensor was designed to distinguish between mass and viscoelastic loads. In this view, the fundamental resonance frequency and quality factor were accessed from the damping spectrum. This QCM immunosensor can be probed to classify blood groups A and B.

Berini and coworkers [57] developed long-range surface plasmon waveguide devices for RBC sensing using anti-A IgG as the recognition element. They exposed the sensing surface to all four blood groups (A, B, AB and O) separately, having equal RBC concentrations. The resultant shifts in output power indicated that blood groups B and O did not specifically interact with the anti-A surface. The observed drop in signals for these blood groups was due to simply the residing of RBCs on the surface. However, blood groups A and AB have shown specific binding interaction with anti-A, as indicated by their respective sensor responses. The sensor surface can be regenerated for successive analyses of RBCs, as the sensor response remains the same even after the sixth round of measurements. Furthermore, using binding response and microscopy images, the authors have also shown that it is indeed possible to detect a single cell with an S/N value of 95. The developed sensing setup is useful for RBC A recognition; however, there was no significant differentiation between blood groups A and AB.

### 4.3. Paper-Based Diagnostics for ABO Blood Group Typing

Shen and coworkers [58] developed paper-based diagnostics of eleven clinically-important secondary BGs by monitoring hemagglutination patterns. They observed that the types of antibodies, their interaction time with RBCs and the pH of the washing buffer are important for the identification of blood groups. The authors used confocal microscopy to study the agglutination pattern mechanism at the cellular level. The results obtained from the developed paper-based devices exhibited a 100% match with the gel card technique for secondary BGs. In another report [59], a paper-based assay for blood group typing was developed, and the results were validated. In this method, a small volume, *i.e.*, 3 µL, blood sample was introduced on the porous paper surface that already contained grouping antibodies. Blood groups ABO and RhD can easily and accurately be detected following the agglutination pattern with suitable reproducibility.

**Figure 6:**
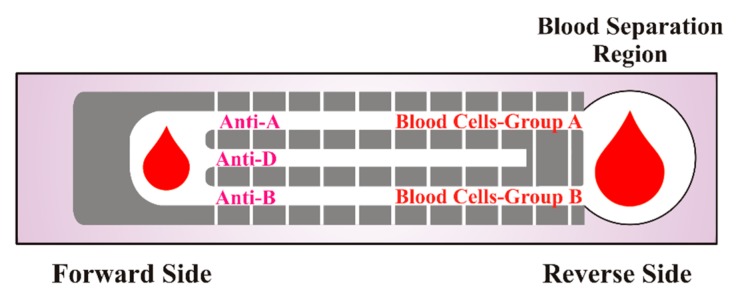
Schematic representation of the paper-based kit for determining Rh, forward and reverse ABO blood groupings; adapted from [60].

Laiwattanapaisal and coworkers [60] reported a paper-based analytical device integrated with immobilized antigens for simultaneous determination of ABO and Rh blood groups. The paper-based kit was made of two different sheets of paper having two different zones. The reverse side has a blood separation zone combined with Whatman paper to separate plasma and serum for reverse grouping. On the forward side of the strip, a special pattern was generated by a wax printer, where the antibodies were fixed. A typical schematic representation of such a paper-based kit is shown in Figure 6.

In order to determine blood type, the ratio of the distance traveled by RBCs to that plasma was calculated. The agglutination pattern on the paper kit indicates the corresponding antibody or antigen type. It was observed that a dilution factor of 1:2 significantly improves the accuracy in forward grouping; however, in the case of reverse grouping, a blood sample without dilution can be used. The authors have shown that paper-based kits can determine ABO and Rh blood groups with accuracy in the range of 85%–90%. Table 1 summarizes the results of paper-based diagnostics of ABO and Rh blood groups. The overall analysis time for blood typing is about 10 min, which is appreciably good for prompt screening. Additionally, the kits used in blood typing can be stored for a week’s time at room temperature if reevaluation is required. Nevertheless, these kits having immobilized antibodies need special storage, *i.e.*, at 4 °C, and can be used up to 21 days. Portability is the main characteristic of the paper-based kits, as they can be used for in-field blood typing, thus making them suitable for point of care applications.

**Table 1:** Paper-based diagnostic results of different blood groups; adapted from [60].

Blood Group	Number of Samples	Accuracy	Discrepancy	Success Rate (%)
**A**	12	11	1	92
**B**	13	11	2	85
**O**	14	13	1	93
**AB**	9	8	1	89
**Rh**	48	46	2	96

Garnier and coworkers [61] reported two different paper-based diagnostic methods for ABO and RhD blood group phenotyping. In the first case, the chromatography principle was followed, where the unbounded blood cells were eluted laterally by washing solvent. The second method utilizes a filtration mechanism where the sample flows through the paper, rather than elution. Although both methods exhibited considerable success for most of the clinically-important blood group phenotypes, however testing for Duffy, MNS (M, N and S antigens present on RBC surfaces) and Lewis groups was ineffective. A comparison of the two methods indicated that the elution-based diagnostic is more suitable for high throughput testing practices, whereas the flow-through method is useful for point of care applications.

### 4.4. Emerging Strategies in Blood Typing

Most of the ABO blood typing techniques are based on specific reactions of antibody-antigens [62] on the erythrocyte surface. There are certain limitations associated with classical methods, which include the availability of rare antiserum, blood typing of recently-transfused patients and for those having a positive anti-globulin test. Moreover, they are not adequate for predicting hemolytic diseases of the fetus. In order to address these issues, molecular blood typing methods [63] are the most suitable ones, as they resolve weak, unexpected and indistinguishable serological findings. Molecular blood typing examined the genetic information in the DNA of the donor or the recipient, which leads to translating the structure of the antigen on the erythrocyte surface. The mechanism is based on the association of the blood cell antigens with a single-nucleotide polymorphism (SNP), which leads to determining the nature of BG antigens on the erythrocyte interface. In this way, BG genotyping methods are established that can be applied to resolve the ambiguous findings of serological methods. They are also useful for patients suffering from autoimmune and hemolytic diseases, transfusion therapy and chemotherapy. Blood group genotyping has been combined with microarray beads for large-scale screening of individuals having rare phenotype blood group antigens. This approach presents a relatively fast and economical method for complete blood genotyping in clinical laboratories.

Polymerase chain reaction with sequence-specific priming (PCR-SSP) is generally applied in transplantation medicine. Recently, PCR-SSP [64] has been adopted to design kits named Conformite Europeenne (CE)-marked test kits for resolving discrepant serological results. These kits works as a supplementary tool along with serological methods; therefore, they cannot be used as the primary technique to replace existing classical methods. There are certain limitations regarding molecular blood typing, as some of the DNA assays do not exclude genes that do not appear in detection, and this often leads to antigen false positives. A Swiss company, Medion Diagnostics, introduced the MD multi-card, which gives fast and safe results for ABO blood typing, as it requires only 100 µL of cell suspension and 300 µL diluent. From one drop of sample, ten different parameters on the test kit can be read within 5 min. Interestingly, it does not require any centrifugation, and the results can be confirmed by validated control spots.

Matrix-assisted laser desorption ionization time of flight mass spectrometry (MALDI-TOF MS) is a modern-day strategy for blood group genotyping. Gassner *et al.* [65] published a comprehensive review article on this topic investigating its potential for accurate and cost-effective high throughput analyses in blood typing.

## 5. Limitations and Challenges

In order to compare the limitations and advantages of both classical and modern trends in blood typing, a table (Table 2) has been drawn. This includes routine serological methods, chemical sensors using both natural and synthetic antibodies and molecular blood typing techniques. It can be seen from this table that most of the classical methods are sensitive, but time consuming, though in certain cases, they are not reliable. In this scenario, molecular techniques can be used as an accompanying tool to make blood typing more accurate. Chemical sensors equipped with blood group-specific antibodies could be a suitable choice for point of care applications; nevertheless, the use of synthetic receptors is more favorable for developing a cost-effective screening system of blood typing.

**Table 2:** A general comparison among some of the classical and modern strategies in blood group typing. SSP, sequence-specific priming; QCM, quartz crystal microbalance.

Test	Principle	Intended for	Analysis Time	Applicability	Remarks
**Slide**	Agglutination	Simple blood group detection	10 min	Hospitals, clinical laboratories	Least sensitive, but low cost; requires small volume; useful for rapid results
**Tube**	Agglutination	Blood group detection and antibody screening	10–30 min	Hospitals, clinical laboratories	Intermediate sensitivity and time consuming
**Microplate**	Agglutination	Blood group detection and antibody screening	10–30 min	Hospitals, clinical laboratories	Fast and highly sensitive
**Column/gel filtration**	Agglutination	Blood group detection and antibody screening	10–45 min	Hospitals, clinical laboratories	Highly sensitive, but time consuming
**Molecular blood typing**	Nucleic acid amplification methods e.g., PCR-SSP	Blood group difficult to identify by serological methods	>1 h	Used as a supplementary tool with classical methods	Highly sensitive, but a lengthy and tedious procedure
**Natural antibody sensors (QCM, SPR)**	Blood group-specific antibodies, e.g., ( IgM) reaction with antigen	Blood group-associated antigens	15–30 min	Limited and confined to research laboratories	Sensitive, but expensive; difficult to regenerate
**Synthetic antibody sensors (QCM)**	Geometrical, as well as chemical adherence of RBCs on a synthetic antibody surface	ABO blood-group typing, sub-blood group detection	10–20 min	Promising, but yet to be established for commercial use	Highly sensitive; easy to regenerate; low cost; useful for several analysis
**Blood test kits**	Lateral assay	Multiparameter identification of blood groups	5 min	Commercially established and validated	Fast and reliable; suitable for emergency diagnosis

One of the main advantages of using synthetic antibodies designed by surface imprinting is their straightforward and relatively easy synthesis schemes, which make them very competitive compared to their natural counterparts. The most striking feature of MIPs is their versatility in bio-recognition, as their surface can be tailored according to the shape and dimension of the target species. Contrarily, the derivation of a typical natural antibody is very tedious and requires many isolation and purification steps, which of course makes it expensive and difficult to obtain. Concerning blood group detection, surface imprinted polymer layers combined with QCM have exhibited suitable potential in developing BG typing sensors. The polymeric receptors not only recognize erythrocytes selectively, but also can differentiate between ABO blood groups in an unambiguous way. Furthermore, they are also capable of distinguishing sub-blood groups A1 and A2, which only differ in surface antigen density. On the other hand, sensors having natural antibodies as receptors are mainly focused on distinguishing between BG A and B only, as experienced from earlier examples. Furthermore, the detection of sub-blood groups by natural receptors is not fully explored. Apart from a detection comparison, the regeneration is a serious concern, since after erythrocyte binding, it is tedious to restore the inherent characteristics of natural antibodies. Even mild washing solvents, like sodium hydroxide, could deteriorate their activity, making them unfit for the next rounds of analyses. Surface imprinted polymers can be used several times with the same efficiency, as the bound erythrocytes can be leached out simply rinsing with deionized water. The number of analyses for blood grouping is directly related to cost, and in this regard, synthetic antibodies are comparatively favorable for using many folds after regeneration. Moreover, unlike natural antibodies, surface imprinted polymers do not require intensive care for storage, since they can be stored in ambient conditions for years with the same affinity features.

There are some concerns associated with the composition of imprinted polymers, as there is no general protocol for their synthesis, including the selection of polymer constituents and optimal polymerization conditions. At the moment, computational modeling and combinatorial strategies for MIP design are also under investigation. However, these approaches are still in their infancy and have to be explored in detail for achieving significant advantages. This is even more important in the case of large bio-macromolecules, because they require both features for suitable recognition, *i.e.*, adequate geometrical and chemical adherence. Although soft lithographic surface imprinting has been established as quite useful, however, it is seldomly studied with combinatorial methods. The combination of both of these could lead to the development of more useful synthetic antibodies for cell recognition. In this way, shape complementary sites with precise chemical interactions, such as van der Waals forces and hydrogen bonding, could be developed.

In hospitals and blood group matching laboratories, there is a need to express the results of the donor or recipient blood groups. Conventional blood matching methods, such as slide or tube tests, require at least 10–30 min for one sample, which is not adequate while handling a large number of samples. Chemical sensors equipped with synthetic antibodies take about 5 min to complete one analysis, *i.e.*, for one BG identification, which is comparatively much better than routine methods. However, developing multi-sensor arrays coated with surface imprinted layers capable of providing necessary information about blood groups would be very useful for high throughput [66] and prompt results. In this perspective, there are two major challenges: one is the optimal design of such a transducer, where each channel gives an independent signal with reduced crosstalk; the second part is optimized synthesis and tailoring of the polymer interface for the intended blood group targets.

Traditional molecular blood group typing methods are useful for routine applications, as they are adequately sensitive and selective; however, in a single assay, it is not possible to get complete information about genetic variants that are related to blood group antigen expression. The established molecular blood typing methods are focused on pre-defined targets, so it is not possible for them to detect new variants. As stated above, more than 300 different blood group antigens are reported, and therefore, regular molecular blood group typing methods need to be revisited for including new genetic variants. Target enrichment next-generation sequencing techniques, such as PCR, hybrid capture enrichment and molecular inversion probes, provide detailed information about genetic sequence and variations.

## 6. Outlook

Traditional blood typing methods are simple, sensitive and reliable, yet time consuming and labor intensive; moreover, the cost of blood group-specific antibodies is quite high. In this respect, synthetic antibodies generated by molecular imprinting are cost effective and offer an expedient way for ABO blood group typing. Along with QCM resonators, these synthetic antibodies can be probed to selectively recognize different ABO blood groups and especially for differentiating between sub-blood groups. Their high selectivity, straightforward regeneration, fast binding kinetics and reusability make them suitable candidates in blood group typing. However, molecular blood group typing provides exclusive genetic information about the DNA of the donor or recipient. This indeed is beneficial in order to have more complete information; thus, weakly reactive or altered antigens could also be determined in patients suffering from different conditions. ABO blood group detection kits provide prompt information based on a lateral flow assay; however, this is more useful for emergency diagnosis. In general, molecular imprinting offers convenient and cost-effective method of producing synthetic antibodies for erythrocyte recognition, and chemical sensors integrated with these synthetic receptors are promising. However, classical methods of blood group typing are still considered the most reliable and, therefore, are adopted in clinical laboratories. In the coming years, more efforts and research should be invested in synthetic antibodies made via imprinting and in transducer devices in order to make them a mature diagnostic technology.

## References

[1] Landsteiner K. (1900). Zur Kenntnis der antifermentativen lytischen and agglutinierenden Wirkung des Blutserums and der lymph. Zentralbl. Bakteriol. Parasit. Infekt..

[2] Landsteiner K. (1901). Ueber Agglutinationserscheinungen normalen menschlichen Blutes. Wien. Klin. Wochenschr..

[3] Liu Z., Liu M., Mercado T., Illoh O., Davey R. (2014). Extended blood group molecular typing and next-generation sequencing. Transfus. Med. Rev..

[4] Rowley M., Milkins C., Lewis S.M., Barbara J.B., Imelda B. (2006). Laboratory aspects of blood transfusion. Dacie and Lewis Practical Haematology.

[5] Westhoff C.M., Reid M.E., Hillyer C.D., Silberstein L.E., Ness P.M., Anderson K.C., Roback J.D. (2007). Chapter 6—Abo and related antigens and antibodies. Blood Banking and Transfusion Medicine.

[6] Malomgre W., Neumeister B. (2009). Recent and future trends in blood group typing. Anal. Bioanal. Chem..

[7] Kim D.S., Lee S.H., Ahn C.H., Lee J.Y., Kwon T.H. (2006). Disposable integrated microfluidic biochip for blood typing by plastic microinjection moulding. Lab Chip.

[8] Heucke U.W., Cobet U. (2000). Blood group typing by ultrasound backscattering—Quantitative measurements of agglutinates and their shear-dependent behavior. Instrum. Sci. Technol..

[9] Dolmashkin A.A., Dubrovskii V.A., Zabenkov I.V. (2012). Blood group typing based on recording the elastic scattering of laser radiation using the method of digital imaging. Quantum Electron..

[10] Doubrovski V.A., Dolmashkin A.A. (2010). Human blood group typing based on digital photographs of RBC agglutination process. Opt. Spectrosc..

[11] Karpasitou K., Drago F., Crespiatico L., Paccapelo C., Truglio F., Frison S., Scalamogna M., Poli F. (2008). Blood group genotyping for Jka/Jkb, Fya/Fyb, S/s, K/k, Kpa/Kpb, Jsa/Jsb, Coa/Cob, and Lua/Lub with microarray beads. Transfusion.

[12] Mujahid A., Lieberzeit P.A., Dickert F.L. (2010). Chemical sensors based on molecularly imprinted sol-gel materials. Materials.

[13] Wagner T., Maris R.J., Ackermann H.-J., Otto R., Beging S., Poghossian A., Schöning M.J. (2007). Handheld measurement device for field-effect sensor structures: Practical evaluation and limitations. Sens. Actuators B Chem..

[14] Mujahid A., Afzal A., Glanzing G., Leidl A., Lieberzeit P.A., Dickert F.L. (2010). Imprinted sol–gel materials for monitoring degradation products in automotive oils by shear transverse wave. Anal. Chim. Acta.

[15] Seidler K., Lieberzeit P.A., Dickert F.L. (2009). Application of yeast imprinting in biotechnology and process control. Analyst.

[16] Pichon V., Chapuis-Hugon F. (2008). Role of molecularly imprinted polymers for selective determination of environmental pollutants—A review. Anal. Chim. Acta.

[17] Piletsky S.A., Turner N.W., Laitenberger P. (2006). Molecularly imprinted polymers in clinical diagnostics—Future potential and existing problems. Med. Eng. Phys..

[18] Haupt K., Mosbach K. (1998). Plastic antibodies: Developments and applications. Trends Biotechnol..

[19] Ye L., Mosbach K. (2008). Molecular imprinting: Synthetic materials as substitutes for biological antibodies and receptors. Chem. Mater..

[20] Schirhagl R., Latif U., Dickert F.L. (2011). Atrazine detection based on antibody replicas. J. Mater. Chem..

[21] Dai H., Xiao D., He H., Li H., Yuan D., Zhang C. (2015). Synthesis and analytical applications of molecularly imprinted polymers on the surface of carbon nanotubes: A review. Microchim. Acta.

[22] Sharma P.S., Iskierko Z., Pietrzyk-Le A., D'Souza F., Kutner W. (2015). Bioinspired intelligent molecularly imprinted polymers for chemosensing: A mini review. Electrochem. Commun..

[23] Schirhagl R., Podlipna D., Lieberzeit P.A., Dickert F.L. (2010). Comparing biomimetic and biological receptors for insulin sensing. Chem. Comm..

[24] Piletska E., Piletsky S., Karim K., Terpetschnig E., Turner A. (2004). Biotin-specific synthetic receptors prepared using molecular imprinting. Anal. Chim. Acta.

[25] Shinkai S., Takeuchi M. (2004). Molecular design of synthetic receptors with dynamic, imprinting, and allosteric functions. Biosens. Bioelectron..

[26] Haupt K., Mosbach K. (2000). Molecularly imprinted polymers and their use in biomimetic sensors. Chem. Rev..

[27] Whitcombe M.J., Chianella I., Larcombe L., Piletsky S.A., Noble J., Porter R., Horgan A. (2011). The rational development of molecularly imprinted polymer-based sensors for protein detection. Chem. Soc. Rev..

[28] Yin J., Yang G., Chen Y. (2005). Rapid and efficient chiral separation of nateglinide and its l-enantiomer on monolithic molecularly imprinted polymers. J. Chromatogr. A.

[29] Xu Z.X., Gao H.J., Zhang L.M., Chen X.Q., Qiao X.G. (2011). The biomimetic immunoassay based on molecularly imprinted polymer: A comprehensive review of recent progress and future prospects. J. Food Sci..

[30] Sellergren B., Allender C.J. (2005). Molecularly imprinted polymers: A bridge to advanced drug delivery. Adv. Drug Deliver. Rev..

[31] Lieberzeit P., Dickert F. (2008). Rapid bioanalysis with chemical sensors: Novel strategies for devices and artificial recognition membranes. Anal. Bioanal. Chem..

[32] Wulff G. (1995). Molecular imprinting in cross-linked materials with the aid of molecular templates—A way towards artificial antibodies. Angew. Chem. Int. Ed..

[33] Defreese J.L., Katz A. (2005). Synthesis of a confined class of chiral organic catalysts via bulk imprinting of silica. Chem. Mater..

[34] Ge Y., Turner A.P.F. (2008). Too large to fit? Recent developments in macromolecular imprinting. Trend. Biotechnol..

[35] Hayden O., Dickert F.L. (2001). Selective microorganism detection with cell surface imprinted polymers. Adv. Mater..

[36] Nishino H., Huang C.-S., Shea K.J. (2006). Selective protein capture by epitope imprinting. Angew. Chem. Int. Ed..

[37] Qin D., Xia Y., Whitesides G.M. (2010). Soft lithography for micro- and nanoscale patterning. Nat. Protoc..

[38] Lieberzeit P.A., Gazda-Miarecka S., Halikias K., Schirk C., Kauling J., Dickert F.L. (2005). Imprinting as a versatile platform for sensitive materials—Nanopatterning of the polymer bulk and surfaces. Sens. Actuators B Chem..

[39] Mujahid A., Iqbal N., Afzal A. (2013). Bioimprinting strategies: From soft lithography to biomimetic sensors and beyond. Biotechnol. Adv..

[40] Dickert F.L., Lieberzeit P., Gazda-Miarecka S., Halikias K., Mann K.-J. (2004). Modifying polymers by self-organisation for the mass-sensitive detection of environmental and biogeneous analytes. Sens. Actuators B Chem..

[41] Dickert F.L., Lieberzeit P., Miarecka S.G., Mann K.J., Hayden O., Palfinger C. (2004). Synthetic receptors for chemical sensors—Subnano- and micrometre patterning by imprinting techniques. Biosens. Bioelectron..

[42] Lieberzeit P., Glanznig G., Jenik M., Gazda-Miarecka S., Dickert F., Leidl A. (2005). Softlithography in chemical sensing—Analytes from molecules to cells. Sensors.

[43] Alizadeh T. (2010). Comparison of different methodologies for integration of molecularly imprinted polymer and electrochemical transducer in order to develop a paraoxon voltammetric sensor. Thin Solid Films.

[44] Jenik M., Seifner A., Krassnig S., Seidler K., Lieberzeit P.A., Dickert F.L., Jungbauer C. (2009). Sensors for bioanalytes by imprinting—Polymers mimicking both biological receptors and the corresponding bioparticles. Biosens. Bioelectron..

[45] Dickert F., Hayden O. (2002). Bioimprinting of polymers and sol-gel phases. Selective detection of yeasts with imprinted polymers. Anal. Chem..

[46] Haupt K., Noworyta K., Kutner W. (1999). Imprinted polymer-based enantioselective acoustic sensor using a quartz crystal microbalance. Anal. Commun..

[47] Dickert F.L., Lieberzeit P., Hayden O. (2003). Sensor strategies for microorganism detection—From physical principles to imprinting procedures. Anal. Bioanal. Chem..

[48] Hayden O., Mann K.-J., Krassnig S., Dickert F.L. (2006). Biomimetic ABO blood-group typing. Angew. Chem. Int. Ed..

[49] Seifner A., Lieberzeit P., Jungbauer C., Dickert F.L. (2009). Synthetic receptors for selectively detecting erythrocyte ABO subgroups. Anal. Chim. Acta.

[50] Brouard D., Ratelle O., Perreault J., Boudreau D., St-Louis M. (2015). Pcr-free blood group genotyping using a nanobiosensor. Vox Sang..

[51] Nanameki K., Ushijima H., Akase S., Matsumoto T., Kamata S. (1999). The rapid measurement of ABO blood type by using surface-plasmon resonance sensor. Bunseki Kagaku.

[52] Quinn J.G., O'Kennedy R., Smyth M., Moulds J., Frame T. (1997). Detection of blood group antigens utilising immobilised antibodies and surface plasmon resonance. J. Immunol. Methods.

[53] Kimura S., Yurugi K., Segawa H., Kuroda J., Sato K., Nogawa M., Yuasa T., Egawa H., Tanaka K., Maekawa T. (2005). Rapid quantitation of immunoglobulin G antibodies specific for blood group antigens a and b by surface plasmon resonance. Transfusion.

[54] Stussi G., Huggel K., Lutz H.U., Schanz U., Rieben R., Seebach J.D. (2005). Isotype-specific detection of abo blood group antibodies using a novel flow cytometric method. Brit. J. Haematol..

[55] Hayashiba K., Sakai M., Kamio K., Hayashiba Y., Tazaki M. (2001). Identification of blood by using quartz-crystal microbalance covered with antibody IgG. Bull. Fac. Eng. Kyushu Sangyo Univ..

[56] Tessier L., Patat F., Schmitt N., Lethiecq M., Frangin Y., Guilloteau D. (1994). Significance of mass and viscous loads discrimination for an at-quartz blood group immunosensor. Sens. Actuators B Chem..

[57] Krupin O., Wang C., Berini P. (2014). Selective capture of human red blood cells based on blood group using long-range surface plasmon waveguides. Biosens. Bioelectron..

[58] Li M., Then W.L., Li L., Shen W. (2014). Paper-based device for rapid typing of secondary human blood groups. Anal. Bioanal. Chem..

[59] Al-Tamimi M., Shen W., Zeineddine R., Tran H., Garnier G. (2012). Validation of paper-based assay for rapid blood typing. Anal. Chem..

[60] Noiphung J., Talalak K., Hongwarittorrn I., Pupinyo N., Thirabowonkitphithan P., Laiwattanapaisal W. (2015). A novel paper-based assay for the simultaneous determination of Rh typing and forward and reverse ABO blood groups. Biosens. Bioelectron..

[61] Then W., Li M., McLiesh H., Shen W., Garnier G. (2015). The detection of blood group phenotypes using paper diagnostics. Vox Sang..

[62] Knowles S., Regan F., Lewis S.M., Barbara J.B., Imelda B. (2006). Blood cell antigens and antibodies: Erythrocytes, platelets, and granulocytes. Dacie and Lewis Practical Haematology.

[63] Westhoff C.M., Beth H.S., Christopher D.H., Charles S.A., Mikhail R. (2013). Molecular DNA based blood group typing. Transfusion Medicine and Hemostasis.

[64] Prager M. (2007). Molecular genetic blood group typing by the use of PCR-SSP technique. Transfusion.

[65] Gassner C., Meyer S., Frey B.M., Vollmert C. (2013). Matrix-assisted laser desorption/ionisation, time-of-flight mass spectrometry–based blood group genotyping—The alternative approach. Transfus. Med. Rev..

[66] Hashmi G., Shariff T., Seul M., Vissavajjhala P., Hue-Roye K., Charles-Pierre D., Lomas-Francis C., Chaudhuri A., Reid M.E. (2005). A flexible array format for large-scale, rapid blood group DNA typing. Transfusion.

